# The Importance of Using Public Health Impact Criteria to Develop Environmental Health Indicators: The Example of the Indoor Environment in New Zealand

**DOI:** 10.3390/ijerph15081786

**Published:** 2018-08-20

**Authors:** Kylie Mason, Kirstin Lindberg, Deborah Read, Barry Borman

**Affiliations:** Environmental Health Indicators Programme, Massey University, Wellington Campus, P.O. Box 756, Wellington 6140, New Zealand; k.lindberg@massey.ac.nz (K.L.); d.read@massey.ac.nz (D.R.); b.borman@massey.ac.nz (B.B.)

**Keywords:** environmental health indicators, environmental burden of disease, attributable burden, public health impact

## Abstract

Developing environmental health indicators is challenging and applying a conceptual framework and indicator selection criteria may not be sufficient to prioritise potential indicators to monitor. This study developed a new approach for prioritising potential environmental health indicators, using the example of the indoor environment for New Zealand. A three-stage process of scoping, selection, and design was implemented. A set of potential indicators (including 4 exposure indicators and 20 health indicators) were initially identified and evaluated against indicator selection criteria. The health indicators were then further prioritised according to their public health impact and assessed by the five following sub-criteria: number of people affected (based on environmental burden of disease statistics); severity of health impact; whether vulnerable populations were affected and/or large inequalities were apparent; whether the indicator related to multiple environmental exposures; and policy relevance. Eight core indicators were ultimately selected, as follows: living in crowded households, second-hand smoke exposure, maternal smoking at two weeks post-natal, asthma prevalence, asthma hospitalisations, lower respiratory tract infection hospitalisations, meningococcal disease notifications, and sudden unexpected death in infancy (SUDI). Additionally, indicators on living in damp and mouldy housing and children’s injuries in the home, were identified as potential indicators, along with attributable burden indicators. Using public health impact criteria and an environmental burden of disease approach was valuable in prioritising and selecting the most important health impacts to monitor, using robust evidence and objective criteria.

## 1. Introduction

Environmental health indicators (EHIs) monitor how the environment affects human health and can be defined as “an expression of the link between environment and health, targeted at an issue of specific policy or management concern and presented in a form that facilitates interpretation for effective decision-making” [1]. When well designed and constructed, these indicators can provide valuable information for policymakers and decisionmakers to prioritise and evaluate actions, as well as raise awareness of issues [2]. However, developing environmental health indicators can be challenging. Indicators must reflect known causal relationships between the environment and health outcomes and meet a range of selection criteria to be useful, reliable, and robust [3]. Little guidance is available on how to develop country-specific EHIs, in particular, when numerous potential indicators meet the selection criteria and need to be prioritised to a set of core indicators.

The main guidance for developing environmental health indicators comes from documents produced by the World Health Organization (WHO) in the 1990s and early 2000s [3], including through its work in the WHO European Region [4] and on children’s environmental health indicators [2]. Additionally, other substantial projects have developed environmental health indicators or health indicator sets with an environmental aspect, including the CHILD project of child health indicators for European countries [5,6], and indicators for children’s health and the environment in North America [7]. In addition to outlining a methodology, these projects developed generic sets of environmental health indicators for use in multiple countries. However, environmental health issues can differ markedly from country to country [3] and depend on the local context. Additionally, new scientific evidence has become available on the links between the environment and health. As a result, environmental health indicators may need to be reviewed or developed by countries, using the most recent evidence and tailored to the country’s needs.

A specific methodology for developing environmental health indicators is outlined in the WHO children’s environmental health indicators report, incorporating the three stages of scoping, selection, and design [2]. The scoping stage involves identifying the important environmental health issues, the key users and their needs, and the information needs that the indicators need to fulfil. The selection stage involves developing a conceptual framework, identifying potential indicators, applying a set of selection criteria to potential indicators, and developing a provisional indicator list. The third stage, design, involves completing detailed indicator design, including assessing data availability, and deciding analysis outputs (for example, time periods and geographic areas).

Ideally, environmental health indicators cover aspects of both environmental exposures and the related health effects to create a balanced set of indicators. Developing a conceptual framework is important for understanding the pathways through which the environment affects human health and identifying what needs to be monitored. A number of conceptual frameworks exist [8], including the DPSEEA (driving forces, pressures, state, exposure, effects, and actions) and more recently, the MEME (multiple exposures, multiple effects) model. The MEME model is based on the DPSEEA model but combines all types of exposures (proximal and distal) and also acknowledges the many-to-many relationships between exposures and health impacts [2]. Regardless of the conceptual model used, health indicators must be based on known (or plausible) causal relationships, that is, an implied exposure-response relationship [2]. Potential indicators are then identified for the different parts of the conceptual framework.

Selecting indicators involves assessing potential indicators against a range of selection criteria. A number of different sets of selection criteria for indicators exist, but the WHO children’s environmental health indicators suggest the following selection criteria [2]: being credible (based on a known linkage), sensitive to change, consistent and comparable over space and time, robust, representative, accurate (based on reliable data), and able to be used at different scales. Indicators should also have clear relevance and utility, be relevant to an issue of policy or practical concern, actionable (related to an issue that is amenable to influence or control), understandable, timely, specific (targeted at an explicit phenomenon or issue), measurable, and cost-effective to produce and use. In New Zealand, the national statistics office has published a similar set of indicator criteria for official statistics, which includes data availability, scientific validity, sensitivity, consistency, comparability, methodologically sound measurements, intelligible and easily interpreted, able to be disaggregated, and timely [9].

Although a provisional list of indicators may sufficiently meet the selection criteria, the list may still need further refinement. A long list of indicators may be resource-intensive to monitor and difficult for users to use and interpret, resulting in requests for a ‘core’ set of indicators [2]. The final set of indicators must also be balanced and represent the conceptual framework [2] rather than place disproportionate emphasis on one part of the framework. Conversely, the indicators should not be over-simplified; they still need to measure the most important issues and be useful and informative [2]. A further process for prioritising potential indicators may therefore be required to complete the selection phase.

Other than applying indicator selection criteria, there is limited guidance for prioritising indicators. One approach is to use working groups and expert advisory groups, such as the WHO approach, with experts screening indicators according to policy relevance, health relevance, and potential data availability [10]. In the context of developing children’s environmental health indicators, Briggs suggested that some indicators may be prioritised if they contain more information than others, for example, by relating to several different issues or by being sufficiently generic to have general applicability [2]. Additionally, the children’s environmental health indicators used burden of disease statistics to prioritise the health needs most important to children globally [2]; similarly, the CHILD project included consideration of the total health burden (on individuals, families, and society) as part of the selection criteria for health indicators [6]. However, only a portion of the full burden of disease will be attributable to environmental factors, and the burden will vary by country. These global statistics are of little help without incorporating additional information such as the health burden within a specific country and the proportion of the health burden attributable to an environmental exposure.

To further prioritise potential indicators, two complementary approaches are helpful. Health impact assessment tools are used to assess the potential health impacts of new policies, programmes, and projects, and they consider the overall public health impacts, as well as health equity, as part of the assessment. Additionally, environmental burden of disease studies (or ‘attributable burden of disease’ studies) use robust evidence about the causal links between environmental exposures and health effects and provide information on the deaths, hospitalisations, and/or healthy years of life lost (measured in disability-adjusted life years (DALYs)) that could have been prevented if people were not exposed to a specific risk factor. These two methodologies were included in a review of potential conceptual frameworks for climate change EHIs [8]. They were rejected as conceptual frameworks, as they were not designed to develop environmental health indicators, and they did not describe the exposure pathway sufficiently [8]. However, we consider that these methodologies have great merit and utility in helping to prioritise indicators as part of a wider indicator development process, including a focus on health equity.

During this project, we developed a new approach for prioritising potential environmental health indicators, based on their public health impact and incorporating an environmental burden of disease approach. This approach was applied during the development of indoor environment EHIs for New Zealand, which covered aspects of housing quality and indoor air quality.

## 2. The Context

The New Zealand Environmental Health Indicators programme is funded by the Ministry of Health to achieve the following:

- to monitor trends in the state of the environment;
- to monitor trends in health outcomes linked to environmental hazards and exposures;
- to compare the environmental health status of geographic areas and population groups;
- to monitor the effectiveness of policies and other interventions on environmental health;
- to help raise awareness about environmental health issues; and
- to help initiate further investigations into links between the environment and health.

As at 2018, the indicator programme monitored over 60 indicators in nine domains: air quality, drinking-water quality, recreational water quality, ultraviolet (UV) exposure, transport, indoor environment, hazardous substances, climate change, border health, and population vulnerability. The indicators are updated annually where possible, and the indicators, factsheets, metadata, and background information are published on the Environmental Health Indicators website (www.ehinz.ac.nz), with additional supporting data about environmental health and the health status of New Zealanders published on an online atlas (www.healthspace.ac.nz).

For this project, we focused on developing indicators for the indoor environment. The indoor environment can affect health in a number of ways, including household crowding, second-hand smoke exposure, cold, damp, and mouldy housing, unflued gas heaters and open fires, safety hazards, lead-based paint flakes, asbestos, and pest infestations [11]. In New Zealand, a key issue is housing availability and affordability, with the recent lack of housing availability and affordability leading to reported overcrowding and homelessness [12]. Additionally, many houses (particularly older houses) have historically been cold and damp, in part due to lack of or poor insulation [13].

Initially, our indoor environment domain only included indicators on household crowding, second-hand smoke exposure, and lack of home heating. A wide range of health outcomes had been identified as being linked to these exposures, but an indicator development process was required to develop a full set of indicators of environmental exposures and related health outcomes.

## 3. Methods

**Figure 1:**
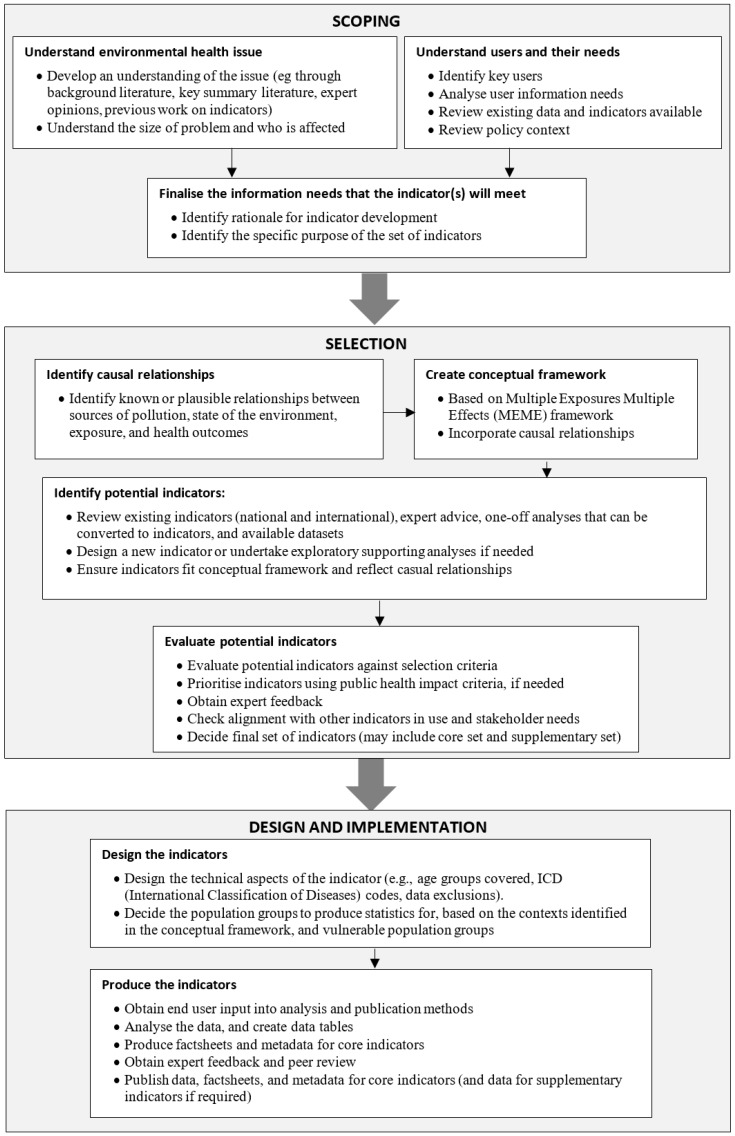
Process for developing a set of environmental health indicators. Source: Adapted from Briggs [2].

To develop New Zealand-specific indicators for the indoor environment, we made some adaptations to the previously mentioned three-stage indicator development process of scoping, selection, and design, outlined in the children’s environmental health indicators report [2] (Figure 1).

### 3.1. Scoping Stage

During the scoping stage, we developed an understanding of the issues relating to the indoor environment and health, using background literature (including key summary documents), subject-matter experts, and previous work on relevant indicators. We identified the links between the indoor environment and health with the most robust scientific evidence of causality, primarily using the WHO assessments of the environmental burden of disease associated with inadequate housing [11] and second-hand smoke exposure [14]. Potential environmental health topics relating to the indoor environment were also identified in the WHO environmental health indicator guidance documents [2,10]. The latest statistics and environmental burden of disease estimates for these exposures in New Zealand were then identified from the literature, including those published by government agencies and academic researchers.

The key users of these indicators were identified as government agencies, including the Ministry of Health, the health sector, local health authorities (e.g., District Health Boards and their public health units), and local councils (territorial authorities). User needs for the environmental health indicators were identified in an earlier stage of the process, in a consultation process with key Ministry of Health staff, and other technical advisors for the establishment of the wider EHI programme.

### 3.2. Selection Stage

We developed a conceptual framework for how the indoor environment affects human health based on the evidence of causation, using the MEME model. Health effects linked to the exposures were identified, based on specific exposure–response relationships from the environmental burden of disease assessments. A distinction was made between health conditions with sufficient evidence of causality (Level 1) and those where the evidence of causality was less convincing but strongly suggestive (Level 2) [14] as determined by teams of experts (e.g., WHO, United States Surgeon General) through meta-analyses and large-scale reviews.

A list of potential indicators was then developed, based on the conceptual framework, and informed by a review of existing indicators (national and international), expert advice, one-off analyses, and available datasets. The indicators identified focused specifically on the environmental exposures and associated health effects (Level 1) identified in the literature as being causally related; the potential exposure and health indicators were then selected to best match those used in the epidemiological evidence. We then identified possible data sources for each potential indicator. Data sources included published statistics, as well as administrative health data from hospitalisations, mortality, and notifiable disease notifications.

Indicators were then evaluated using indicator selection criteria based on Statistics New Zealand’s good practice guidelines for indicator development [9] (Table 1). The assessment was then peer-reviewed by public health experts and people with experience and knowledge of the datasets.

### 3.3. Assessing the Public Health Impact of Potential Indicators

To further prioritise the potential health indicators, we introduced an additional criterion, ‘public health impact’, which was defined as “Indicator needs to relate to an environmental health issue of significant public health impact to New Zealand. This health impact may include affecting a large number of people, a vulnerable population, or Māori health [the indigenous people of New Zealand]; or having substantial policy relevance”. The public health impact of each potential health indicator was assessed based on five sub-criteria: the number of people affected, the severity of health impact, whether vulnerable population groups were affected or large inequalities were apparent, whether the indicator related to multiple environmental exposures, and whether the indicator had policy relevance (Table 2). This criterion and its sub-criteria were adapted from the New Zealand health impact assessment tool for assessing the potential health impacts of new policies and projects [15]. For each health indicator, the assessment of the public health impact used the health condition data most aligned to the potential indicator.

To assess the number of people affected, we used published New Zealand figures on the burden of disease attributable to second-hand smoke [16] and household crowding [17], by disease. For the burden of asthma attributable to damp and mouldy housing, no recent estimates were available for New Zealand. To estimate the attributable burden of disease, we calculated the population attributable fraction (PAF), which is the proportion of cases of a disease that are attributable to a risk factor. The PAF is given by the following formula:

$$\mathrm{PAF}=\frac{p\left(RR-1\right)}{p\left(RR-1\right)+1}$$

where *p* is the prevalence of exposure in the population, and *RR* is the relative risk (or odds ratio, which is an estimate of the relative risk from certain types of studies). A relative risk of 1.56 (95% confidence interval 1.30–1.86) was used for current asthma for children exposed to dampness and mould [18] and an estimated prevalence of exposure to dampness and mould in the home of 31.8% in adults from the 2014/2015 New Zealand General Social Survey [19]. This gave an approximate PAF of 15%. The attributable burden of disease was estimated by multiplying the total burden of disease by the PAF:

Attributable burden=PAF×burden of disease

We used published figures of 3552 hospitalisations due to asthma in children aged 0–14 years in 2015 [20], from which to estimate the attributable burden in children.

For injuries occurring in the home, no PAFS were available, so the total number of deaths and hospitalisations were used. We used the most recent available data, from 1989–1998, for injuries in children aged 0–4 years [21]. However, incompleteness of data for the location of injury in the hospitalisations dataset is likely to have underestimated these numbers.

The attributable burdens were tabulated in the published measurement unit, for example, attributable deaths, hospitalisations, or DALYs (which combine years of life lost (YLL) to premature death, and years of life lived in disability (YLD) due to illness or injury).

### 3.4. Finalising the Set of Environmental Health Indicators to Monitor

Based on the results of the indicator selection criteria and public health impact criteria assessments, we grouped indicators that met the criteria into the following:

1. Core set of indicators: the most important indicators to monitor to be produced annually, with factsheets, webpages, and data in an online atlas;
2. Indicators for further investigation: indicators that may become core indicators but need further work and/or data to become available before a final decision is made; and
3. Attributable burden indicators: to be produced occasionally (for example, every 3–5 years) to update the burden of disease attributable to a specific environmental exposure.

### 3.5. Design and Implementation Stage

In the final design stage, technical details of core indicators were finalised. For indicators requiring analysis of datasets, the design stage included defining ICD (International Classification of Diseases) codes, age groups, and exclusions (for example, hospitalisations excluded hospital transfers, day cases, overseas visitors, and deaths). This work drew heavily on other published indicators and methodologies used in the New Zealand context, as identified in the scoping stage. Analyses of confidentialised unit record files were carried out using SAS software, version 9.4 (SAS Institute Inc., Cary, NC, USA), and statistical outputs of health indicators included crude and age-standardised rates, and 95% confidence intervals [22]. Analyses were output for the total population, and by sex, age group, prioritised ethnic group, New Zealand Index of Deprivation quintiles (NZDep2013) [23], urban–rural status, health districts (District Health Boards), and local councils (territorial authorities). These population groups included some vulnerable groups identified in the scoping stage. Metadata (indicator profiles) were produced for all indicators to give details of the design.

## 4. Results

### 4.1. Scoping Stage

Household crowding, second-hand smoke exposure, damp and mouldy housing, and unsafe homes were identified as major exposure issues relating to the indoor environment in New Zealand. A review of the literature identified that household crowding affected 16% of children aged 0–14 years and 10% of the total population in New Zealand in 2013 [24]. An estimated 1300 hospital admissions for infectious diseases were attributable to household crowding in New Zealand (average annual numbers for 2007–2011) [17], with children and Māori and Pacific peoples most affected [17]. Additionally, second-hand smoke exposure in the home affected 5.0% of children aged 0–14 years in 2012/2013 and 3.7% of non-smoking adults [25]. An estimated 104 deaths were caused by second-hand smoke exposure in New Zealand in 2010, as well as the loss of 2286 healthy years of life (DALYs) in 2006, with males, children, and Māori disproportionately affected [16]. Furthermore, in 2014/2015 an estimated 31.8% of New Zealand adults reported living in a house with dampness or mould [19]. While the attributable burden due to dampness and mould has not been estimated for New Zealand, a review estimated that 16–28% of current asthma in New Zealand children and adults was caused by this risk factor in 2000 [26]. Lastly, childhood injuries occurring in the home led to 390 deaths and 24,635 hospitalisations in children aged 0–4 years over the ten years, 1989–1998 [21].

### 4.2. Identifying Causal Relationships

**Figure 2:**
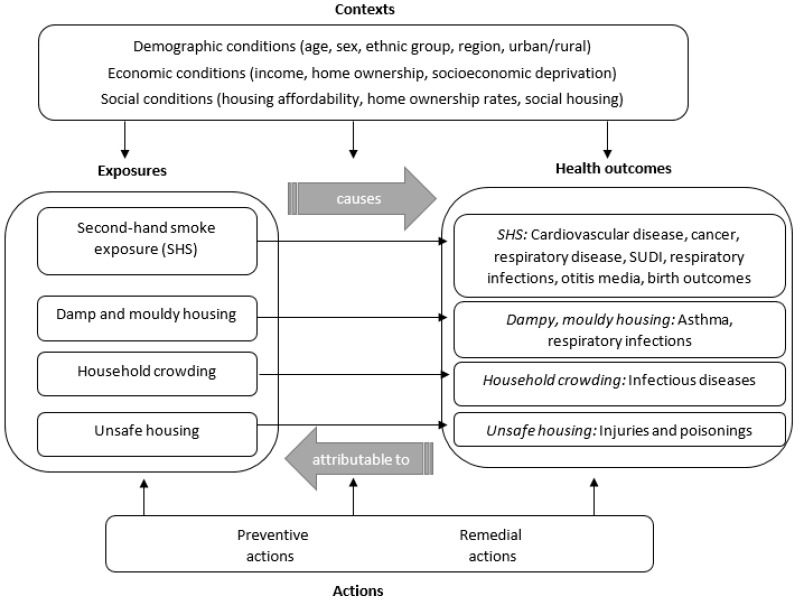
The multiple exposures multiple effects (MEME) framework, applied to the indoor environment. Source: Adapted from Briggs [2]. SUDI: sudden unexpected death in infancy.

A conceptual model using the MEME framework was developed, incorporating the four selected exposures and their related health effects (Figure 2). A full range of the health impacts caused by the environmental exposures were then identified from the literature (Table 3). For household crowding, a recent meta-analysis identified the following health effects as being causally related to household crowding: hepatitis A and *Helicobacter pylori* infection in all ages, tuberculosis in adults, and gastroenteritis, pneumonia/lower respiratory tract infection, bronchiolitis, *Haemophilus influenzae* type b (Hib disease), and meningococcal disease in children [17]. Additionally, upper respiratory tract infections were found to be strongly associated with household crowding for children, while some evidence supported links with acute rheumatic fever and serious skin infections.

For second-hand smoke exposure in the home, eight conditions were identified as being caused by second-hand smoke exposure [14,27,28,29], including lung cancer, ischaemic heart disease, and stroke in non-smoking adults, and asthma, lower respiratory tract infections, and otitis media in children. Additionally, having a mother that smoked in the first year of life increased the risk of sudden unexpected death in infancy (SUDI), while having a non-smoking mother exposed to second-hand smoke during pregnancy increased the risk of the new-born being small for gestational age. Other health impacts with level 2 evidence (suggesting causality) included meningococcal disease in children [30,31] and pre-menopausal female breast cancer [32], chronic obstructive pulmonary disease [33], and asthma in non-smoking adults [28].

For damp and mouldy housing, indoor coldness and excess moisture can lead to mould growth, which has been linked to respiratory problems [34,35]. Recent reviews identified asthma exacerbation in children as being the only outcome with sufficient evidence to deduce causation [26,34]. For unsafe home environments, the children’s environmental health indicator work [2] was used to identify physical injuries in the home (particularly falls and burns) and poisonings in the home for children as key health outcome indicators.

### 4.3. Identifying and Evaluating Potential Indicators against Indicator Selection Criteria

Using the environmental exposures and health impacts identified in Table 3, 24 potential indicators (4 exposure indicators and 20 health indicators) were identified, and possible data sources identified. The majority of these data sources were government-collected datasets, including the New Zealand Health Survey, the Census, and administrative health datasets.

A total of 19 of the 24 potential indicators met all the Statistics New Zealand selection criteria (Table 4). Of the five indicators that did not meet the all the criteria, three were health effects from second-hand smoke exposure in non-smoking adults. These health effects are difficult to monitor among non-smokers only; additionally, the lag time between exposure and the health effects reduces the indicator’s sensitivity to change, and the overall impact that second-hand smoke exposure has on these diseases is small (PAF of about 2% in non-smokers).

Several potential sources of data were identified for the indicator of living in damp and mouldy housing: the New Zealand General Social Survey, which asked adults about living in damp and mouldy houses, and the 2018 New Zealand Census of Populations and Dwellings, which collected similar data on living in damp and mouldy housing for the total population, as well as data on houses without a heating source. Given that children are the highest priority population group based on the health evidence, and that measuring damp and mouldy housing directly was preferable to using a proxy about homes without a heating source, the Census data on damp and mouldy housing was selected as the most appropriate data source.

For *Helicobacter pylori* infection, little prevalence or incidence data were available for New Zealand. A possible alternative was to monitor hospitalisations for sequelae of *H. pylori* infection (non-cardia gastric cancer, peptic ulcer, gastritis, and duodenitis) [3]. However, not all cases of *H. pylori* infection result in sequelae [36]. Additionally, *H. pylori* is typically acquired during childhood and does not usually resolve spontaneously, so there can be a large lag time between exposure and health effects [36].

### 4.4. Assessing the Public Health Impact

The public health impact criteria were then applied to all the potential health indicators to prioritise the most important indicators to monitor from a public health perspective (Table 5). The health impacts with the highest burden due to second-hand smoke exposure included ischaemic heart disease, stroke, lung cancer, and SUDI. For household crowding, pneumonia/lower respiratory tract infections (including bronchiolitis) had the highest burden. For damp and mouldy housing, asthma had a large burden in children, while for unsafe housing, there were almost 2500 hospitalisations annually for injuries in the home among children aged 0–4 years in 1989–1998.

In terms of severity of health impact, SUDI had the most severe impact (death of a baby), while some other health conditions could also cause death (ischaemic heart disease, stroke, lung cancer, meningococcal disease, and unintentional injury). Some diseases could lead to long-term illness and disability, including asthma and meningococcal disease. While inequalities were apparent for most health conditions, asthma, lower respiratory tract infections, and meningococcal disease had high inequalities, particularly for Māori and/or Pacific peoples. These health conditions also related to multiple environmental exposures.

To some degree, all indicators were related to environmental issues of policy relevance, with either the potential for policy actions and/or the issue being of current policy interest. Housing quality, household crowding, and children’s health are of particular policy interest at present in New Zealand [12]. Additionally, an epidemic of meningococcal disease in the 1990s led to a vaccination campaign in New Zealand, so future monitoring is important in case of another outbreak. Tuberculosis was less important from an environmental health policy perspective, as historically most cases of tuberculosis in New Zealand have been due to importation [37].

### 4.5. Finalising the Set of Environmental Health Indicators for the Indoor Environment

Based on the indicator selection process and public health impact assessment, eight core indicators were selected: living in crowded households, exposure to second-hand smoke in the home, maternal smoking at two weeks post-birth, asthma prevalence, asthma hospitalisations, lower respiratory tract infection hospitalisations, SUDI, and meningococcal disease notifications (Table 6). These indicators were selected as they met the indicator selection criteria and also had a substantial public health impact, in particular, meeting multiple sub-criteria. For example, the lower respiratory tract infection hospitalisations indicator was selected as it related to both household crowding and second-hand smoke exposure, had considerable health burden as a result of these, and disproportionately affected some vulnerable population groups. The definition for this indicator also included bronchiolitis and Hib disease, so the wider condition group of lower respiratory tract infections was selected for monitoring.

In addition to the eight core indicators, an indicator on living in damp and mouldy housing was identified to be developed when the 2018 Census data becomes available. An indicator about children’s injury occurring in the home was also identified for further investigation to assess the quality of injury location data in the hospitalisation dataset and to determine the specific types of injuries to monitor (for example falls, burns, and poisoning). Developing these indicators would ensure a more balanced and cohesive set of indicators for monitoring the indoor environment in New Zealand.

To complement the core indicators, we also decided to include three attributable burden indicators: the attributable health burden due to second-hand smoke exposure; infectious disease hospitalisations attributable to household crowding; and children’s asthma burden attributable to damp and mouldy housing. These indicators would include an overall assessment of the health impacts of each environmental exposure, incorporating all health effects with Level 1 evidence of causality, as well as up-to-date information on population exposure levels and the PAF.

### 4.6. Designing the Final Set of Indicators

The final stage of developing the core set of indicators involved specifying the definitions for each indicator, including ICD codes, age groups for monitoring, and hospital exclusions. These details, and the latest results for each indicator (including for selected vulnerable populations), are provided in Table 6. For the indicators from the National Minimum Dataset and EpiSurv, the results are based on analysis of the confidentialised datasets.

## 5. Discussion

### 5.1. The Importance of Using a ‘Public Health Impact’ Approach to Develop Environmental Health Indicators

This project has demonstrated the importance and value of using a public health impact approach when developing environmental health indicators and has provided a framework and methodology that can be used for other topics and in other countries to prioritise and select environmental health indicators. Using public health impact criteria and an attributable burden of disease approach during the indicator selection process ensures that environmental health indicators are based on robust, scientific evidence about the health burden caused by the environmental exposure and therefore focus on the most important health needs of the country. A focus on policy relevance, vulnerable populations, and the number of people affected ensures that indicators are relevant and useful to decisionmakers and policymakers, who can use policies, programmes, and projects to address these environmental health issues.

This work also demonstrates how prevalence data about exposure in the population can be used to estimate the proportion of health burden attributable to an environmental exposure, through the population attributable fraction (PAF). In this paper, we demonstrated this method by estimating the PAF for children’s asthma caused by damp and mouldy housing in New Zealand at 15%. Calculating the PAF allows the development of ‘attributable burden indicators’, which have been suggested as the most robust form of environmental health indicators [Briggs, personal communication], as they link specifically between the environmental exposure and the related health effects.

Using a public health impact criterion and sub-criteria extends previously published processes for developing environmental health indicators [2,3,4,10]. Researchers and developers of indicators may have intuitively used a similar process in the past for prioritising environmental health indicators. For example, a WHO working group screened potential indicators based on their ‘policy relevance, health relevance and potential data availability’ [43]. The public health impact criteria are also similar to criteria suggested in the children’s environmental health indicators (‘relevant to an issue of policy or practical concern’ and ‘actionable’) [2]. However, our approach goes further than these criteria, as it includes an assessment of the health impact from the environmental exposure for the country of interest and gives researchers a solid and systematic framework for carrying out an assessment. Having a set of specific public health impact criteria, against which to assess indicators, moves decision making from intuition to a transparent and informed decision.

We have also highlighted some modifications to the WHO approach for developing environmental health indicators, which are useful when developing indicators for a specific country. Most importantly, it is beneficial to identify data sources before applying the indicator selection criteria, rather than in the design stage, to ensure that the proposed indicators meet selection criteria such as data availability, timeliness, consistency, comparability, and methodologically-sound measurement. Additionally, identifying causal relationships in the selection stage ensures that the conceptual framework is based on robust evidence and, furthermore, that both health and exposure indicators monitor outcomes for the most important age groups. A further addition to the WHO approach was to identify vulnerable population groups in the scoping stage and to consider these groups in both the selection and design stages of indicator development.

Overall, we found that using a public health impact approach was valuable for identifying a ‘core set’ of indicators for regular monitoring. While our approach has only been applied to health indicators at this stage, the approach could also be adapted for exposure variables as well. We also identified attributable burden indicators for potential development: the burden of disease attributable to second-hand smoke exposure, household crowding, and damp and mouldy housing. These indicators may require more work than other indicators and so would be updated less regularly than the core indicators. However, the attributable burden indicators are beneficial for informing action, as they estimate the potential health impacts that could be gained from removing the environmental exposure. For example, eliminating household crowding in New Zealand would prevent an estimated 1300 hospitalisations for infectious diseases each year. Thus, attributable burden indicators show how improving a specific aspect of the environment would have substantial health benefits.

### 5.2. Findings from the Indoor Environment Indicators

Our indoor environment indicators showed that the indoor environment has a sizeable impact on health in New Zealand. In particular, damp and mouldy housing has a relatively large burden on children’s health in New Zealand, accounting for an approximate 500 hospitalisations for asthma each year. Future work and upcoming Census data releases will help to refine this estimate, as well as provide a better estimate of the percentage of children living in damp and mouldy houses and the population attributable fraction. Household crowding affected almost one in six (16%) children in New Zealand in 2013, increasing their risk of infectious diseases; the recent housing affordability issues in New Zealand [12] may well have impacted on this figure, and upcoming data from the 2018 Census may shed more light on this issue. By comparison, second-hand smoke exposure affected 5% of children and 3.7% of non-smoking adults in New Zealand; nonetheless, the health impacts from second-hand smoke exposure were significant, particularly for babies exposed to second-hand smoke from their mother in their first year, which increases the risk of SUDI.

Focusing on vulnerable population groups was an important part of this indicator development process. Potentially vulnerable populations were identified at the scoping stage and were considered as part of the public health impact assessment. This information then informed how indicator results were output to ensure monitoring of high priority population groups. In particular, our indicators showed that some ethnic groups were disproportionately affected by the indoor environment in New Zealand. For example, household crowding affected 43% of Pacific children and 25% of Māori children in 2013. These high exposure rates were mirrored by higher rates of lower respiratory tract infection hospitalisations and meningococcal disease notifications for children in these ethnic groups. This example demonstrates the power of having several health effect indicators relating to one environmental exposure to allow triangulation of health impacts. Additionally, focusing on vulnerable population groups provides evidence to inform targeted actions to help reduce inequalities.

### 5.3. Limitations of Our Approach

A limitation of this study was the comparison of published attributable burdens using different measurement units (attributable deaths, hospitalisations, and DALYs). DALYs tend to be the most helpful measurement, as they combine both fatal outcomes (years of life lost to premature death) and non-fatal outcomes (years of life lived in disability or ill-health). While it is not ideal to compare across exposures using different measurements, we were able to use a consistent measurement within each individual exposure, which allowed the most important health effects for each exposure to be identified. Additionally, the attributable burden data were only used as a guide for prioritising indicators and using the published statistics was a pragmatic decision given limited resources. A helpful next step could be converting the various attributable burdens into the same measurement, for example, by applying PAFs to country-specific DALYs sourced from the Global Burden of Disease Study (http://ghdx.healthdata.org/) or a similar national study. The Global Burden of Disease Study is also a useful data source for deaths and DALYs at the country level when other data sources or research results are unavailable.

An additional limitation was the lack of published PAFs for the injury indicator, which meant we were unable to estimate the proportion of injuries attributable to an unsafe indoor environment. The total number of injuries occurring in the home for 0–4-year-olds was used instead in the public health impact assessment; while this may be an over-estimate of the attributable burden, it still provided useful information about the total injury burden occurring in the indoor environment to aid indicator selection.

One of the drawbacks of the prioritisation process was that the final core set of indicators did not include some health conditions with large attributable burdens, for example, ischaemic heart disease and stroke, which mainly affect older adults. These indicators were ruled out based on the indicator selection criteria, including difficulties in monitoring these conditions in non-smokers, lag times of 1–5 years between exposure and health impact, and a small PAF of 2%. In particular, a small PAF indicates that only a small fraction of the overall health burden is attributable to the environmental exposure, resulting in the potential indicator being less sensitive to change and therefore less valid. Nonetheless, these health conditions, and other potential indicators identified through the selection process, could be considered worthy of monitoring. There are two potential ways of addressing these limitations. Firstly, having attributable burden indicators ensure that all health conditions and age groups are included in the assessment of health impact, as part of the environmental burden of disease methodology. Secondly, a set of supplementary indicators can be developed as part of regular monitoring. This would allow lower priority health indicators to still be monitored, although not to the same level of detail or with the same amount of commentary as the core set of indicators. These supplementary indicators would also be informative for other areas of environmental health monitoring, such as outdoor air quality. For the indoor environment, potential supplementary indicators could include ischaemic heart disease, stroke, lung cancer, gastroenteritis hospitalisations, otitis media hospitalisations (acute admissions, and waiting list for grommet insertion), bronchiolitis from respiratory syncytial virus (RSV) infection, hospitalisations for sequelae of *H. pylori* infection, and tuberculosis notifications. Additionally, health indicators with Level 2 evidence of causality (such as upper respiratory tract infections) could also be included as supplementary indicators if desired. These supplementary indicators could also be output by different population groups to enable monitoring of vulnerable population groups (such as children and older adults).

More generally, one limitation of using a public health impact approach is that it does not provide the answer to what indicators to select. However, this approach still helps inform the final decision and provides a set of criteria to guide the decision-making process. In particular, this approach allows the indicator developers to communicate and discuss the merits of each indicator and moves the decision-making to a transparent and more systematic process.

Our assessment of indoor environment indicators for New Zealand was limited to four main exposures but could have included a wide range of additional exposures. These include indoor cold (separate from dampness and mould), traffic noise, and exposure to substances, such as lead, carbon monoxide, and formaldehyde [11]. Other aspects of the indoor environment, such as indoor radon and indoor cooking on open wood or coal fires, have little relevance in New Zealand and are not considered national environmental health issues of concern.

## 6. Conclusions

This study has demonstrated a novel and valuable approach for prioritising and selecting environmental health indicators based on their ‘public health impact’. The public health impact of potential indicators was assessed based on five sub-criteria: the number of people affected (based on environmental burden of disease statistics); severity of health impact; whether vulnerable populations were affected and/or large population inequalities were apparent; whether the indicators relate to multiple environmental exposures; and relevance to policy. The approach can be used when numerous possible health indicators have been identified and need to be reduced to a ‘core’ set of indicators. In this way, this approach fills a gap by describing how to move from the results of applying indicator selection criteria to a final list of indicators. Moreover, we propose some modifications to the indicator development method described in the children’s environmental health indicators to tailor the process for a specific country.

After following the indicator development process, the following core indicators were selected to monitor the indoor environment in New Zealand: living in crowded households, exposure to second-hand smoke, maternal smoking at two weeks after birth, asthma prevalence, asthma hospitalisations, lower respiratory tract infection hospitalisations, meningococcal disease notifications, and sudden unexpected death in infancy (SUDI). Additionally, indicators on living in damp and mouldy housing and children’s injuries in the home were identified for further development, subject to data availability and quality. Furthermore, we identified indicators of the attributable health burden due to household crowding, second-hand smoke exposure, and damp and mouldy housing.

These indicators, selected using a public health impact approach, showed that housing quality and availability has a sizeable impact on the health of New Zealanders, particularly for children and Māori and Pacific peoples. Addressing these environmental issues would have substantial health benefits for New Zealand and reduce health inequalities for some population groups. Using public health impact criteria and an environmental burden of disease approach proved valuable in identifying and prioritizing the most important environmental health impacts to monitor.

## Figures and Tables

**Table 1:** Indicator selection criteria for New Zealand environmental health indicators.

Indicator Selection Criteria	Explanation
Data availability	Indicator must have data that can be easily and reliably extracted.
Scientifically valid	Indicator must have an established, scientifically sound link to the environmental health issue.
Sensitive	Indicator should respond relatively quickly and noticeably to changes but not show false movements.
Consistent	Indicator should be consistent with those used in other indicator programmes (including internationally) so comparisons can be made.
Comparable	Indicator should be consistent to allow comparisons over time.
Methodologically sound measurement	Indicator measurement needs to be methodologically sound.
Intelligible and easily interpreted	Indicator should be sufficiently simple to be interpreted in practice and be intuitive in the sense that it is obvious what the indicator is measuring.
Able to be disaggregated	Indicator needs to be able to be broken down into population subgroups or areas of particular interest, such as ethnic groups or regional areas.
Timely	Data needs to be collected and reported regularly and frequently to ensure it is reflecting current and not historical trends.

Source: Based on the indicator selection criteria published by the Advisory Committee on Official Statistics [9].

**Table 2:** Public health impact criteria for environmental health indicators.

Public Health Impact Selection Criteria	Explanation
Public health impact	Indicator needs to relate to an environmental health issue of significant public health impact to New Zealand. This health impact may include affecting a large number of people, a vulnerable population, or Māori health; or having substantial policy relevance.
(i) Affecting a large number of people	Priority should be given to health effects affecting larger numbers of people (i.e., with a higher burden of disease attributable to the environmental exposure (e.g., measured as deaths, hospitalisations, or disability-adjusted life years (DALYs))), within the country/area of interest.
(ii) Severity of impact	Priority should be given to health conditions with severe impacts, such as severity of illness, long-term repercussions (such as disability or long-term illness), and/or risk of death.
(iii) Affecting vulnerable populations and/or having large inequalities	Priority should be given to health effects that particularly affect vulnerable population groups and/or have large health inequalities.
(iv) Relating to multiple exposures or health effects	Priority should be given to health indicators that link to multiple environmental exposures.
(v) Relevant to policy	Priority should be given to indicators where there is potential for policy actions about the environmental exposure to make a difference; and/or the issue is of current policy interest.

**Table 3:** Environmental exposures and related health effects, for the indoor environment.

Environmental Exposure	Pathway	People Exposed	Health Effects—Causal (Level 1)	Health Effects—Suggestive (Level 2)
Household crowding	Household crowding	Children living in crowded households	GastroenteritisPneumonia/lower respiratory tract infection Bronchiolitis from respiratory syncytial virus (RSV) infection *Haemophilus influenzae* (Hib disease) Meningococcal disease	Upper respiratory tract infection Serious skin infections Acute rheumatic fever
		People (all ages) living in crowded households	Hepatitis A *Helicobacter pylori* infection Tuberculosis	
Second-hand smoke (SHS) exposure	Maternal smoking and/or exposure to SHS	Babies exposed to SHS through maternal smoking	Sudden unexpected death in infancy (SUDI)	
	Maternal exposure to SHS during pregnancy	Babies exposed to SHS in utero (non-smoking mother exposed to SHS)	Small for gestational age (low birthweight)	Preterm delivery
	Second-hand smoke exposure in the home	Children exposed to SHS in the home	Asthma Lower respiratory tract infection (including bronchitis, bronchiolitis, pneumonia, and acute respiratory infection) Otitis media	Meningococcal disease
		Non-smoking adults exposed to SHS in the home	Ischaemic heart disease Stroke Lung cancer	Asthma (induction, exacerbation) Breast cancer Chronic obstructive pulmonary disease (COPD)
Damp, mouldy, cold houses	Damp and mould	Children living in damp and mouldy houses	Asthma exacerbation	Respiratory infections, bronchitis
		Adults living in damp and mouldy houses		Asthma exacerbation Respiratory infections, bronchitis
Unsafe home environment	Physical hazards in the home	Children	Falls Burns	Drowning
	Hazardous chemicals in the home	Children	Poisonings	

**Table 4:** Potential environmental health indicators identified for the indoor environment (*n* = 24).

Potential Indicator	Data Source	Meet All Criteria?	Indicator Selection Criteria									Comments
			Data Availability	Scientifically Valid	Sensitive to Change	Consistent	Comparable	Methodologically Sound	Intelligible and Easily Interpreted	Able to Be Disaggregated	Timely	
**Exposure indicators**												
People living in crowded households	Census	Yes	√	√	√	√	√	√	√	√	√	Both children and total population are important to monitor.
People exposed to SHS in the home	NZHS	Yes	√	√	√	√	√	√	√	√	√	Both children and adults are important to monitor.
Mothers smoking at two weeks postnatal	Maternity	Yes	√	√	√	√	√	√	√	√	√	
People living in damp and mouldy houses	Census (living in damp and mouldy houses)	No	×	√	√	√	√	√	√	√	√	Data about people living in damp and mouldy houses are not currently available but will be available in 2019 from the 2018 Census. Not known if this data will be collected again.
	NZGSS	No	×	√	√	√	√	√	√	?	?	Only adults are covered, not children. Data collected twice (2010 and 2014)—unknown if it will be collected again.
	Census (no source of home heating)	No	√	×	√	√	√	√	√	√	√	Data about households with no source of home heating could be a proxy for cold houses, which are associated with damp and mouldy housing. However, this is not a good measure of cold houses (for example, some people do not use available home heating due to cost) or damp and mouldy houses and will mis-measure the true value.
**Health indicators**												
Lower respiratory tract infection hospitalisations in children	NMDS	Yes	√	√	√	√	√	√	√	√	√	Evidence for 0–1 years (SHS) and 0–5 years (household crowding)
Bronchiolitis from RSV hospitalisations	NMDS	Yes	√	√	√	√	√	√	√	√	√	Bronchiolitis is also included in the definition for lower respiratory tract infection.
*Helicobacter pylori* infection hospitalisations	NMDS	No	√	√	×	√	√	√	√	√	√	Hospitalisations are for sequelae of *H. pylori* infection, including non-cardia gastric cancer, peptic ulcer, gastritis, and duodenitis. There can be a large lag time between exposure and health effects.
Gastroenteritis hospitalisations	NMDS	Yes	√	√	√	√	√	√	√	√	√	
Tuberculosis hospitalisations	NMDS	Yes	√	√	√	√	√	√	√	√	√	
Meningococcal disease notifications	EpiSurv	Yes	√	√	√	√	√	√	√	√	√	Evidence for 0–16 years (household crowding) and suggested link with SHS
Hepatitis A hospitalisations	NMDS	Yes	√	√	√	√	√	√	√	√	√	
*Haemophilus influenzae* (Hib disease) hospitalisations	NMDS	Yes	√	√	√	√	√	√	√	√	√	One of the ICD-10-AM codes for Hib disease (J14) is also included in the definition for lower respiratory tract infection.
Asthma prevalence in children	NZHS	Yes	√	√	√	√	√	√	√	√	√	Evidence for 0–14 years (SHS, dampness/mould).
Asthma hospitalisations in children	NMDS	Yes	√	√	√	√	√	√	√	√	√	Evidence for 0–14 years (SHS, dampness/mould). Asthma hospitalisations are a proxy for asthma exacerbation.
Sudden unexpected death in infancy (SUDI)	MoH	Yes	√	√	√	√	√	√	√	√	√	Strong evidence for mothers smoking after birth
Otitis media/grommets hospitalisations in children	NMDS	Yes	√	√	√	√	√	√	√	√	√	
Ischaemic heart disease hospitalisations/deaths in non-smoking adults	NMDS/Mort	No	×	√	×	√	√	√	√	√	√	Difficult to get data for non-smokers. Lag-time of 1–5 years after exposure.
Stroke hospitalisations/deaths in non-smoking adults	NMDS/Mort	No	×	√	×	√	√	√	√	√	√	Difficult to get data for non-smokers. Lag-time of 1–5 years after exposure.
Lung cancer registrations/deaths in non-smoking adults	Cancer/Mort	No	×	√	×	√	√	√	√	√	√	Difficult to get data for non-smokers. Lag-time of 10–20 years after exposure.
Small for gestational age (low birthweight)	Maternity	Yes	√	√	√	√	√	√	√	√	√	Data are available for proportion of all babies born at term gestation who are small for their gestational age.
Unintentional injuries in the home in children	NMDS	Yes	√?	√	√	√	√	√	√	√	√	The data exists, but it depends whether location data is robust enough to include. Needs further investigation.
Falls in the home in children	NMDS	Yes	√?	√	√	√	√	√	√	√	√	
Burns in the home in children	NMDS	Yes	√?	√	√	√	√	√	√	√	√	
Poisonings in the home in children	NMDS	Yes	√?	√	√	√	√	√	√	√	√	

Abbreviations: √ = Meets criteria; × = Does not meet criteria; SHS = second-hand smoke; Census = New Zealand Census of Populations and Dwellings; NZHS = New Zealand Health Survey; Maternity = New Zealand Maternity Clinical Indicators; NZGSS = New Zealand General Social Survey; EpiSurv = EpiSurv notifiable disease surveillance database; MoH = Ministry of Health publications; NMDS = National Minimum Dataset (hospitalisations data); Mort = New Zealand Mortality Collection; Cancer = New Zealand Cancer Registry; ICD-10-AM = International Statistical Classification of Diseases and Related Health Problems, Tenth Revision, Australian Modification.

**Table 5:** Assessment of health indicators for indoor environment, by public health impact criteria.

Potential Health Indicator	Health Condition (Relating to Potential Health Indicator)	Age Group for Attributable Burden Evidence	Met All Other Indicator Selection Criteria?	Public Health Impact Criteria						Recommend
				(i) Proportion Attributable (PAF, %)	(i) Attributable Burden	(ii) Severity of Impact	(iv) Vulnerable Populations Affected and/or Inequalities	(iii) Multiple Exposures	(v) Specific Policy Relevance of Indicator	
Household Crowding Indicators				Annual Attributable Hospitalisations (2007–2011) [17]						
Lower respiratory tract infection hospitalisations	Lower respiratory infections/pneumonia	0–5 years	Yes	10%	669	Short-term, rarely fatal	Children, Māori, Pacific	Yes		Core
Bronchiolitis hospitalisations	Bronchiolitis from RSV	0–3 years	Yes	16%	644	Short-term, rarely fatal	Children, Māori, Pacific			
Hospitalisations for sequelae of *Helicobacter pylori* infection	*Helicobacter pylori* infection	0+ years	Yes	8%	102	Long-term, can lead to other health problems; not all *H. pylori* infection leads to health impacts	Māori, Pacific peoples			
Gastroenteritis hospitalisations	Gastroenteritis	0–5 years	Yes	2%	42	Short-term, rarely fatal in New Zealand	Children			
Tuberculosis notifications	Tuberculosis	15+ years	Yes	19%	22	Long-term; takes a long time to treat and cure; can have some sequelae.	Māori adults, Pacific adults		In New Zealand, little disease transmission takes place within-country	
Meningococcal disease notifications	Meningococcal disease	0–16 years	Yes	15%	5	Can be fatal and may cause long-term disability	Children, Māori, Pacific peoples	Yes (second-hand smoke Level 2)	An epidemic in the 2000s led to national vaccination campaign	Core
Hepatitis A hospitalisations	Hepatitis A	0+ years	Yes	5%	1					
Hospitalisations for *Haemophilus influenza* type b	*Haemophilus influenzae* type b	0–6 years	Yes	10%	0.7		Children			
Second-Hand Smoke Exposure Indicators				Attributable DALYs (2006) [16]						
Ischaemic heart disease hospitalisations/deaths in non-smoking adults	Ischaemic heart disease	15+ years non-smokers	No	1.5%	1033	Can be fatal				
Stroke hospitalisations/deaths in non-smoking adults	Stroke	35+ years non-smokers	No	1.3%	389	Can be fatal, cause long-term disability				
Lung cancer registrations/deaths in non-smoking adults	Lung cancer	15+ years non-smokers	No	2.2%	96	Often fatal				
Sudden unexpected death in infancy (SUDI)	SUDI	0 years	Yes	11.3%	596	Fatal	Children, Māori		SUDI prevention activities are funded in New Zealand	Core
Asthma prevalence	Asthma (onset, ever had asthma)	0–14 years	Yes	3.1%	93	Long-term, rarely fatal	Children, Māori	Yes		Core
Lower respiratory tract infection hospitalisations	Lower respiratory tract infections	0–1 years	Yes	3.1%	42	Short-term, rarely fatal	Children, Māori	Yes		Core
Otitis media hospitalisations (acute; grommets)	Otitis media	0–14 years	Yes	2.6%	31	Short-term	Children, Māori			
Small for gestational age	Low birthweight at term	0 years	Yes	2.5%	6	Can lead to long-term effects	Children, Māori			
Damp and Mouldy Housing Indicator				Annual Attributable Hospitalisations (2015)						
Asthma hospitalisations	Asthma exacerbation	0–14 years	Yes	15%	537	Long-term illness	Children, Māori, Pacific	Yes		Core
Unsafe Environments Indicator				Annual Impacts (1989–1998) [21]						
Unintentional injuries hospitalistations (for injuries occurring in the home)	Unintentional injuries in the home	0–4 years	Yes	Not available	39 deaths; 2464 hospital admissions (total numbers)	Can be fatal, may cause long-term disability	Children			Core, but subject to further investigation of data quality for location data

**Table 6:** Final set of core environmental health indicators for indoor environment.

Indicator	Age Group	Data Source	Design Details	Latest Results for New Zealand (Year of Data)
**Exposures**				
Proportion of people living in crowded households	0–14 years, Total population	Census	People living in a house requiring one or more additional bedrooms, according to the Canadian National Occupancy Standard [24]	10% of total population; 16% of children; 25% of Māori children; and 43% of Pacific children (2013)
Proportion of children and non-smoking adults exposed to second-hand smoke in the home	0–14 years 15+ years	New Zealand Health Survey	People reporting that someone smoked inside the house [25]	5.0% of children; 3.7% of non-smoking adults; and 9.2% of Māori children (2012/13)
Mothers smoking at two weeks postnatal	All mothers who gave birth in that year	New Zealand Maternity Clinical Indicators	Mothers who reported that they smoked at two weeks after birth, among all mothers who reported a smoking status at two weeks after birth [38]	12% of mothers; and 32% of Māori mothers (2015)
**Health Effects**				
Prevalence of asthma in children	2–14 years	New Zealand Health Survey publications	Children aged 2–14 years who have been diagnosed by a doctor as having asthma, and who are currently using inhalers, medicine, tablets, pills, or other medication for it [39]	16.6% of children; 24.0% of Māori children; and 17.4% of Pacific children (2015/2016)
Asthma hospitalisations in children	0–14 years	National Minimum Dataset	Acute and semi-acute hospitalisations with asthma (ICD-10AM J45–J46) or wheeze (R06.2) as the primary diagnosis, for children aged 0–14 years. Analyses excluded overseas visitors, deaths, and transfers within and between hospitals. Wheeze is included as there is evidence that paediatricians are more likely to diagnose suspected asthma as wheeze for younger children in New Zealand [40,41].	682 hospitalisations per 100,000 children; 838 per 100,000 (Māori); and 1324 per 100,000 (Pacific) (2016)
Lower respiratory tract infection hospitalisations in children	0–4 years	National Minimum Dataset	Acute and semi-acute hospitalisations with pneumonia (ICD-10AM J12–J16, J18), bronchitis (J20), bronchiolitis (J21) or unspecified acute lower respiratory tract infection (J22) as the primary diagnosis, for children aged 0–4 years.	3050 hospitalisations per 100,000 children; 4254 per 100,000 (Māori); and 6711 per 100,000 (Pacific) (2016)
Meningococcal notifications	0–14 years	EpiSurv	Notifications of meningococcal disease, in children aged 0–14 years.	35 notifications; highest rates in Māori and Pacific children (2016)
Sudden unexpected death in infancy (SUDI)	0 years	Fetal and infant deaths publication	Deaths in children aged under one year of age (<1 year old) with an underlying cause of death in the following ICD-10AM codes: R95, R96, R98, R99, W75, W78, W79. Rates are presented per 1000 live births [42].	45 deaths; highest rate in Māori babies (2014)

## References

[1] Corválan C., Briggs D.J., Kjellstrom T., Briggs D.J., Corválan C., Nurminen M. (1996). Development of environmental health indicators. Linkage Methods for Environment and Health Analysis. General Guidelines.

[2] Briggs D.J. (2003). Making a Difference: Indicators to Improve Children’s Environmental Health.

[3] World Health Organization (1999). Environmental Health Indicators: Framework and Methodologies.

[4] World Health Organization (2004). Development of Environment and Health Indicators for European Union Countries: Results of a Pilot Study: Report On a WHO Working Group Meeting, Bonn, Germany, 7–9 July 2004.

[5] Rigby M. (2005). Principles and challenges of child health and safety indicators. Int. J. Inj. Control Saf. Promot..

[6] Rigby M., Köhler L. (2002). Child Health Indicators of Life and Development (CHILD): Report to the European Commission.

[7] Commission for Environmental Cooperation (2006). Children’s Health and the Environment in North America: A First Report on Available Indicators and Measures.

[8] Hambling T., Weinstein P., Slaney D. (2011). A review of frameworks for developing environmental health indicators for climate change and health. Int. J. Environ. Res. Public Health.

[9] Advisory Committee on Official Statistics (2009). Good Practice Guidelines for the Development and Reporting of Indicators.

[10] World Health Organization (2000). Environmental Health Indicators: Development of a Methodology for the WHO European Region.

[11] Braubach M., Jacobs D., Ormandy D. (2011). Environmental Burden of Disease Associated with Inadequate Housing.

[12] Johnson A., Howden-Chapman P., Eaqub S. (2018). A Stocktake of New Zealand’s Housing.

[13] Buckett N., Marston N., Saville-Smith K., Jowett J., Jones M. (2011). Preliminary BRANZ 2010 Housing Condition Survey Report.

[14] Öberg M., Jaakkola M.S., Prüss-Üstün A., Schweizer C., Woodward A. (2010). Second-Hand Smoke: Assessing the Burden of Disease at National and Local Levels.

[15] Public Health Advisory Committee (2005). A Guide to Health Impact Assessment: A Policy Tool for New Zealand.

[16] Mason K., Borman B. (2016). Burden of disease from second-hand smoke exposure in New Zealand. N. Z. Med. J..

[17] Baker M., McDonald A., Zhang J., Howden-Chapman P. (2013). Infectious Diseases Attributable to Household Crowding in New Zealand: A Systematic Review and Burden of Disease Estimate.

[18] Fisk W.J., Lei-Gomez Q., Mendell M.J. (2007). Meta-analyses of the associations of respiratory health effects with dampness and mold in homes. Indoor Air.

[19] Statistics New Zealand Perceptions of Housing Quality in 2014/15. http://archive.stats.govt.nz/browse_for_stats/people_and_communities/housing/perceptions-housing-quality-2014-15.aspx.

[20] Telfar Barnard L., Zhang J. (2016). The Impact of Respiratory Disease in New Zealand: 2016 Update.

[21] Gulliver P., Dow N., Simpson J. (2005). The epidemiology of home injuries to children under five years in New Zealand. Aust. N. Z. J. Public Health.

[22] Eayres D. (2008). Commonly Used Public Health Statistics and Their Confidence Intervals: Technical Briefing 3.

[23] Atkinson J., Salmond C., Crampton P. (2014). NZDep2013 Index of Deprivation.

[24] Statistics New Zealand (2018). Living in a Crowded House: Exploring Ethnicity and Well-Being of People in Crowded Households.

[25] Ministry of Health (2014). Tobacco Use 2012/13: New Zealand Health Survey.

[26] Prezant B., Douwes J. (2011). Calculating the Burden of Disease Attributable to Indoor Dampness in New Zealand. http://www.academia.edu/27232468/Calculating_the_burden_of_disease_attributable_to_indoor_dampness_in_NZ.

[27] U.S. Department of Health and Human Services (2014). The Health Consequences of Smoking—50 Years of Progress. A Report of the Surgeon General.

[28] U.S. Surgeon General (2006). The Health Consequences of Involuntary Exposure to Tobacco Smoke—A Report of the Surgeon General.

[29] Cal-EPA (2005). Proposed Identification of Environmental Tobacco Smoke as a Toxic Air Contaminant.

[30] Lee C.C., Middaugh N.A., Howie S.R., Ezzati M. (2010). Association of secondhand smoke exposure with pediatric invasive bacterial disease and bacterial carriage: A systematic review and meta-analysis. PLoS Med..

[31] Murray R.L., Britton J., Leonardi-Bee J. (2012). Second hand smoke exposure and the risk of invasive meningococcal disease in children: Systematic review and meta-analysis. BMC Public Health.

[32] Johnson K.C., Miller A.B., Collishaw N.E., Palmer J.R., Hammond S.K., Salmon A.G., Cantor K.P., Miller M.D., Boyd N.F., Millar J. (2011). Active smoking and secondhand smoke increase breast cancer risk: The report of the Canadian expert panel on tobacco smoke and breast cancer risk (2009). Tob. Control.

[33] Eisner M.D., Balmes J., Katz P.P., Trupin L., Yelin E.H., Blanc P.D. (2005). Lifetime environmental tobacco smoke exposure and the risk of chronic obstructive pulmonary disease. Environ. Health.

[34] Kanchongkittiphon W., Mendell M.J., Gaffin J.M., Wang G., Phipatanakul W. (2015). Indoor environmental exposures and exacerbation of asthma: An update to the 2000 review by the institute of medicine. Environ. Health Perspect..

[35] Palaty C., Shum M. (2012). Health Effects from Mould Exposure or Dampness in Indoor Environments.

[36] BPAC (2014). The Changing Face of Helicobacter pylori Testing.

[37] Das D., Baker M., Venugopal K., McAllister S. (2006). Why the tuberculosis incidence rate is not falling in New Zealand. N. Z. Med. J..

[38] Ministry of Health (2018). New Zealand Maternity Clinical Indicators 2016.

[39] Ministry of Health (2016). Annual Update of Key Results 2015/16: New Zealand Health Survey.

[40] HQSC (2016). Atlas of Healthcare Variation Methodology: Asthma.

[41] Simpson J., Duncanson M., Oben G., Adams J., Wicken A., Butchard M., Pierson M., Lilley R., Gallagher S. (2016). The Health Status of Children and Young People in New Zealand 2015.

[42] Ministry of Health (2017). Fetal and Infant Deaths 2014.

[43] Pond K., Kim R., Carroquino M.-J., Pirard P., Gore F., Cucu A., Nemer L., MacKay M., Smedje G., Georgellis A. (2007). Workgroup report: Developing environmental health indicators for European children: World Health Organization working group. Environ. Health Perspect..

